# Hypoxic Treatment Decreases the Physiological Action of the Herbicide Imazamox on *Pisum sativum* Roots

**DOI:** 10.3390/plants9080981

**Published:** 2020-08-03

**Authors:** Miriam Gil-Monreal, Mercedes Royuela, Ana Zabalza

**Affiliations:** Institute for Multidisciplinary Research in Applied Biology (IMAB), Universidad Publica de Navarra, Campus Arrosadia s/n, 31006 Pamplona, Spain; mirian.gil@unavarra.es (M.G.-M.); royuela@unavarra.es (M.R.)

**Keywords:** acetolactate synthase, ethanol fermentation, imidazolinones, mode of action, aerobic fermentation

## Abstract

The inhibition of acetolactate synthase (ALS; EC 2.2.1.6), an enzyme located in the biosynthetic pathway of branched-chain amino acids, is the target site of the herbicide imazamox. One of the physiological effects triggered after ALS inhibition is the induction of aerobic ethanol fermentation. The objective of this study was to unravel if fermentation induction is related to the toxicity of the herbicide or if it is a plant defense mechanism. Pea plants were exposed to two different times of hypoxia before herbicide application in order to induce the ethanol fermentation pathway, and the physiological response after herbicide application was evaluated at the level of carbohydrates and amino acid profile. The effects of the herbicide on total soluble sugars and starch accumulation, and changes in specific amino acids (branched-chain, amide, and acidic) were attenuated if plants were subjected to hypoxia before herbicide application. These results suggest that fermentation is a plant defense mechanism that decreases the herbicidal effect.

## 1. Introduction

Imazamox (IMX) is an imidazolinone herbicide that inhibits acetolactate synthase (ALS), also known as acetohydroxy acid synthase (EC 2.2.1.6), the enzyme that catalyzes the condensation of either two molecules of pyruvate to form acetolactate or one molecule of pyruvate and one molecule of 2-ketobutyrate to form 2-aceto-2-hydroxybutyrate in the biosynthetic pathway of branched-chain amino acids (BCAAs): leucine, isoleucine, and valine [1].

ALS-inhibiting herbicides emerged in the 1980s and they have been demonstrated as potent, selective, broad-spectrum herbicides. These chemicals are the largest site-of-action group on the market, with more than 50 active ingredients belonging to five classes (imidazolinones, sulfonylureas, triazolopyrimidines, pyrimidinyl(thio)benzoates, and sulfonylamino-carbonyl triazolinones). Altogether, ALS-inhibiting herbicides accounted for approximately 15% of the total herbicide market in 2015 [2].

Although the biochemical mechanisms underlying the blocking of ALS activity through ALS inhibitors has been studied [3], less is known regarding their modes of action, which are the physiological processes underlying plant death resulting from inactivated ALS. To understand the physiological effects involved in the lethal process after herbicide treatment is important as it can lead to their more rational use, and because it can help in the development of new compounds with similar herbicidal activities, but with different enzyme inhibition targets to avoid the evolution of weed resistance.

Previous findings showed that ALS inhibitors cause growth arrest followed by the slow death of treated plants [4,5], along with changes in the free amino acid content [6,7] and impairment of carbon metabolism, such as carbohydrate accumulation [8,9,10]. Another effect on the roots of plants treated with ALS inhibitors is the induction of aerobic fermentation and the alternative respiratory pathway; both of which are low-ATP producing pathways [9,11,12,13,14,15]. All these metabolic impairments indicate that the effect of these herbicides on primary plant metabolism has broader physiological consequences than a lack of certain amino acids alone.

Fermentation is an essential pathway activated in plants exposed to hypoxia. Plant hypoxia often originates because of variations in the environment, such as flooding or severe rainfall [16]. Under these circumstances, plants have to adapt their metabolism in order to avoid energy shortage [17]. Induction of the fermentative metabolism allows the plant to use glycolysis for ATP production by recycling NAD^+^ [18]. In the ethanol fermentation pathway, pyruvate decarboxylase (PDC, EC 4.1.1.1) catalyzes the conversion of pyruvate to acetaldehyde, which is then converted to ethanol by the action of the enzyme alcohol dehydrogenase (ADH, EC 1.1.1.1), with the concomitant regeneration of NAD^+^ [18]. Besides its role during oxygen deprivation, ethanol fermentation is induced in response to a number of stressful aerobic conditions, such as osmotic stress or low-temperature conditions [19,20,21,22,23]. It has been suggested that fermentation improves cold stress tolerance [24] and that this pathway might act as an overflow regulating carbohydrate metabolism [25].

Although fermentation in plants exposed to low-oxygen conditions has been studied in depth, its role in plants exposed to other stresses when oxygen is not limited has yet to be fully explained; therefore, the role of fermentation induction in the mode of action of ALS inhibitors remains to be elucidated. Two, non-contradictory explanations can be considered. Firstly, the induction of this pathway could be a plant defense mechanism that promotes better tolerance of the herbicide. Secondly, it could contribute to the chemical’s toxicity, since ethanol and lactate, metabolites shown to be toxic for the plants, are produced during fermentation [18].

The objective of this study was to unravel the importance of ethanol fermentation in plant responses to ALS inhibitors, trying to outline if fermentation induction is related to the toxicity of the herbicides or if it is a plant defense mechanism that alleviates the herbicidal effect. An original experimental design was employed to achieve this aim. Pea plants were exposed to two different times of hypoxia before herbicide application in order to induce the ethanol fermentation pathway prior to ALS inhibition. Then, IMX was applied to the nutrient solution and the characteristic physiological effects triggered by ALS inhibitors were evaluated (carbohydrates and amino acid profile).

## 2. Results

Pea plants were exposed to low-oxygen conditions before the herbicide was applied, thus when the plants were treated with the herbicide, they presented an enhanced fermentative metabolism. Using this approach, the effects of the herbicide could be compared between the plants that demonstrated an induced fermentative metabolism before ALS inhibition, and those in which the fermentation pathway was not activated when the herbicide was applied. Specifically, one group of plants was exposed to 48 h of hypoxia (the Hypoxia-48h group), another group was exposed to 24 h of hypoxia (the Hypoxia-24h group), and a third group was maintained in normal oxygen conditions (the No-Hypoxia group). IMX was applied to half of the plants in each group, while the others were not treated with herbicides and were used as the control for the corresponding group. The different treatments were named with a combination of two codes: the first code refers to the hours of hypoxia (0, 24 h, or 48 h), and the second one indicates if IMX was applied (IMX) or if they were controls (C) (see Table 1 for details): 0-C, 0-IMX, 24h-C, 24h-IMX, 48h-C, 48h-IMX.

### 2.1. Validation of the Experiment

**Figure 1:**
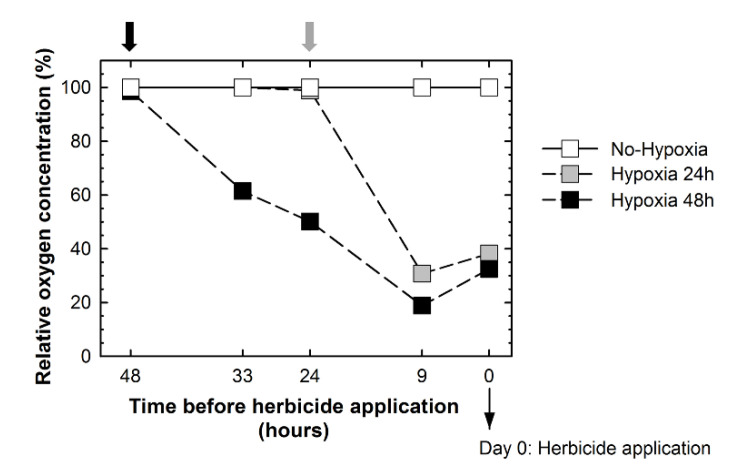
Relative oxygen concentration (%) in the nutrient solution before herbicide application. Black and grey arrows above the graph indicate aeration removal 48 h and 24 h before herbicide application, respectively.

To ascertain that the wanted low-oxygen conditions were obtained, the oxygen concentration in the nutrient solution was monitored (Figure 1). When the aeration was removed (indicated with a black arrow for the Hypoxia-48h group and grey arrow for the Hypoxia-24h group in Figure 1), the oxygen concentration in the nutrient solution drastically decreased. At day 0, the oxygen concentration present in the nutrient solution of the tanks from the Hypoxia-48h and Hypoxia-24h groups was about 30–40%. By contrast, in the tanks that were continuously aerated (the No-Hypoxia group), the oxygen concentration remained at around 100%. These results indicate that the desired conditions to carry out the experiment were obtained.

**Figure 2:**
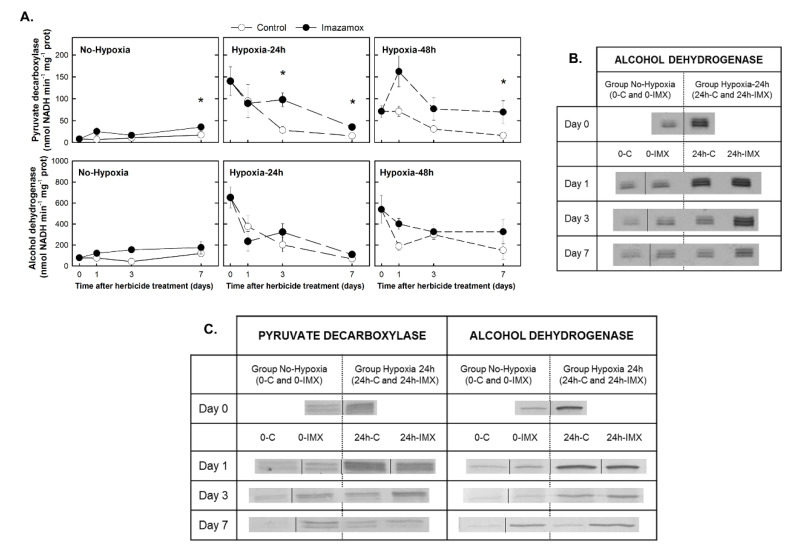
Pyruvate decarboxylase (PDC) and alcohol dehydrogenase (ADH) in the roots of pea plants. Pea plants were not treated with hypoxia before herbicide application (the No-Hypoxia group) or were treated with hypoxia for 24 h or 48 h before herbicide application (the Hypoxia-24h and Hypoxia-48h groups). Half of the plants from each group were treated with imazamox (5 mg L^−1^, black symbols). The others were not treated with herbicide and were used as controls (white symbols). The different treatments were named with a combination of two codes: the first code refers to the hours of hypoxia (0, 24 h, or 48 h), and the second one indicates if plants were treated with imazamox (IMX) or were controls (C): 0-C, 0-IMX, 24h-C, 24h-IMX, 48h-C, 48h-IMX. (**A**) In vitro activities of PDC and ADH in the roots of pea plants. Values represent the mean ± SE (*n* = 4 biological replicates). Significant variations are marked with * for differences between control and imazamox-treated plants (*t*-Test, *p* < 0.05) on a given day. See Supporting Information Table S1 for the two-way ANOVA results. (**B**) Native PAGE for ADH activity in pea roots. Each lane contained 1.75 µg of protein. (**C**) Immunoblot detection of PDC and ADH on days 0, 1, 3, and 7 after imazamox application. Each lane contained 30 µg of protein. In blots and gels, each vertical dividing line indicates lane or lanes removed from the original image. See supporting Information Figures S1, S2 and S3 for full gel images.

The in vitro enzymatic activities of PDC and ADH (Figure 2A) were measured in the roots of the plants from the six studied treatments (Table 1). The herbicide application increased the activity of PDC in the roots of the plants of the No-Hypoxia group. At day 0, the PDC activity detected in the roots of the plants that were exposed to hypoxia was much higher than the activity found in the roots of the plants from the No-Hypoxia group. Once the plants were again aerated, the PDC activity decreased to control values in the plants that were not treated with IMX, while it remained higher in the IMX-treated plants with respect to their controls in the Hypoxia-24h and Hypoxia-48h groups. Similar to the PDC activity, at the beginning of the experiment, the ADH activity was much higher in the groups that were exposed to hypoxia before herbicide application. Once the plants were again aerated, the activity of ADH decreased to control values. In this case, the herbicidal effect in all groups was not very pronounced.

The in gel enzymatic activity of ADH (Figure 2B) and the protein content of the enzymes PDC and ADH (Figure 2C) were determined in the No-Hypoxia and Hypoxia-24h groups. The three ADH isoenzymes described in peas were detected in the native PAGE for ADH activity (from top to bottom: ADH1-ADH1, ADH1-ADH2, and ADH2-ADH2) [26], and the pattern of band intensity (Figure S1) was similar to the in vitro activity. In the No-Hypoxia group, IMX application increased the activity of the ADH1-ADH2 and ADH2-ADH2 isoenzymes from day 3. The PDC and ADH protein content (Figure 2C) showed a similar pattern as the in vitro activity, showing that the increase in activity is due, at least in part, to an increase in the amount of fermentative enzymes.

### 2.2. Growth Parameters

**Figure 3:**
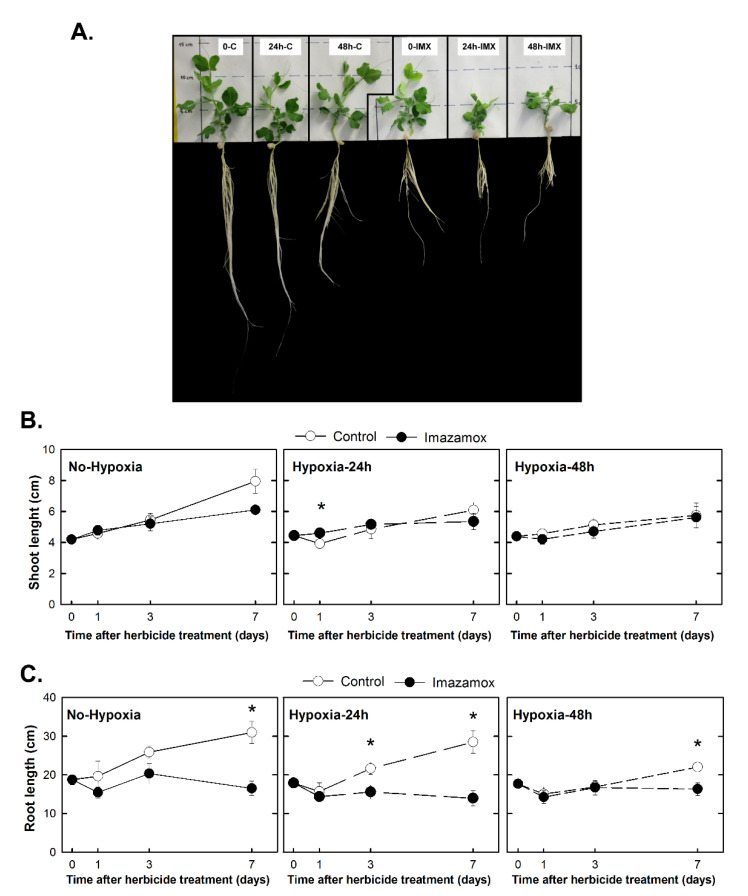
Effect of imazamox on the growth of pea plants. Pea plants were not treated with hypoxia before herbicide application (the No-Hypoxia group) or were treated with hypoxia for 24 h or 48 h before herbicide application (the Hypoxia-24h and Hypoxia-48h groups). Half of the plants from each group were treated with imazamox (5 mg L^−1^, black symbols) and the others were not treated with herbicide and were used as controls (white symbols). The different treatments were named with a combination of two codes: the first code refers to the hours of hypoxia (0, 24 h, or 48 h), and the second one indicates if plants were treated with imazamox (IMX) or they were controls (C): 0-C, 0-IMX, 24h-C, 24h-IMX, 48h-C, 48h-IMX. (**A**) Aspect of the plants 7 days after herbicide application. (**B**) Shoot length (**C**) Root length. Values represent the mean ± SE (*n* = 4 biological replicates). Significant variations are marked with * for differences between control and imazamox-treated plants (*t*-Test, *p* < 0.05) on a given day. See Supporting Information Table S1 for the two-way ANOVA results.

In order to study the effect of IMX on the growth of the different studied groups, the shoot and root lengths were measured (Figure 3). Figure 3A shows the aspect of the plants 7 days after herbicide application. The roots of the IMX-treated plants became brownish while the roots of the control plants were white. The growth of the secondary roots was inhibited and the growth of the shoot and the principal root was also arrested by the herbicides.

The shoot of the control plants from the Hypoxia-24h and Hypoxia-48h groups grew less than the plants from the No-Hypoxia group (Figure 3B), indicating that hypoxia had an effect on shoot growth. The effect of IMX on the shoot growth of the treated plants was similar in the three groups, regardless of whether they were previously exposed to hypoxia. Root growth was arrested in the No-Hypoxia group as a consequence of herbicide application after 3 and 7 days of treatment (Figure 3C). The effect of the herbicide in the Hypoxia-48h group was not so evident because the roots of the control plants (48h-C) practically did not grow until the third day.

### 2.3. Carbohydrate Content

**Figure 4:**
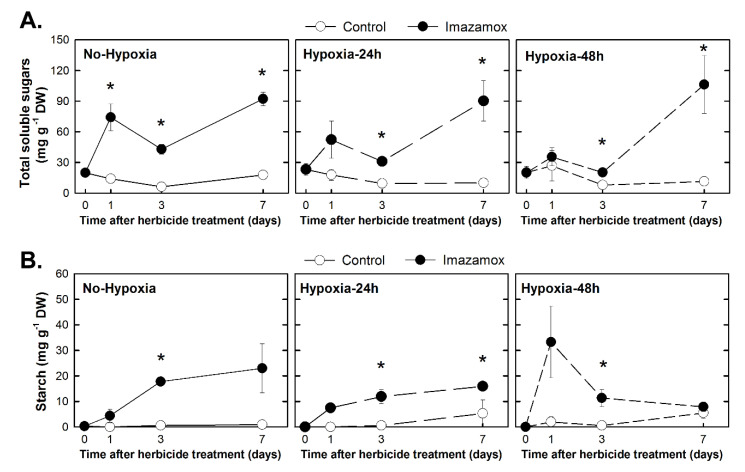
Total soluble sugars (**A**) and starch (**B**) content in the root of pea plants. Pea plants were not treated with hypoxia before herbicide application (the No-Hypoxia group) or were treated with hypoxia for 24 h or 48 h before herbicide application (the Hypoxia-24h and Hypoxia-48h groups). Half of the plants from each group were treated with imazamox (5 mg L^−1^, black symbols). In each group, the others were not treated with herbicide and were used as the controls (white symbols) for the imazamox-treated plants. Values represent the mean ± SE (*n* = 4 biological replicates). Significant variations are marked with * for differences between control and imazamox-treated plants (*t*-Test, *p* < 0.05) on a given day. See Supporting Information Table S1 for the two-way ANOVA results.

The sum of sucrose, fructose, glucose (Figure 4A), and starch (Figure 4B) contents were measured in the roots of the pea plants. Total soluble sugars were accumulated in the herbicide-treated plants from all the studied groups. While the effect of the herbicide on the non-hypoxic plants was significant as early as day 1 of the IMX treatment, the significant effect on the 24h-IMX and 48h-IMX plants was delayed until the third day of treatment.

Starch was also accumulated in the roots of the plants of the three studied groups as a consequence of IMX application (Figure 4B). The plants exposed to hypoxia before herbicide application showed lower starch accumulation at day 7 than the plants that were not exposed to hypoxia (Figure 4B).

### 2.4. Free Amino Acid Profile

**Figure 5:**
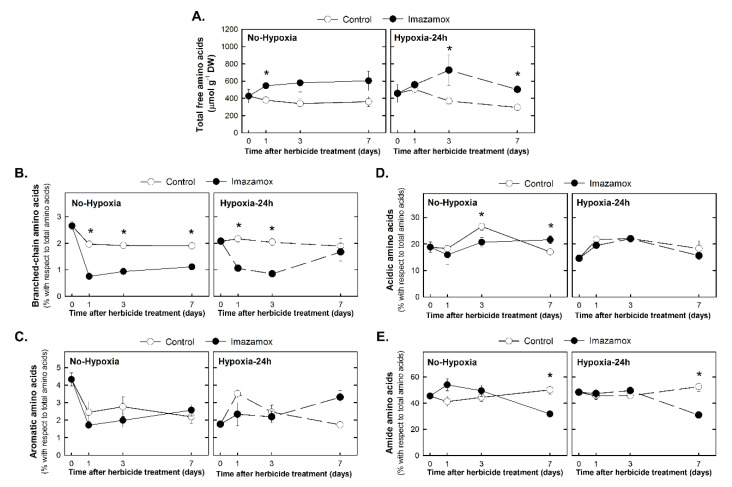
Free amino acid profile in the root of pea plants. Pea plants were not treated with hypoxia before herbicide application (Group No-Hypoxia) or were treated with hypoxia for 24 h before herbicide application (Group Hypoxia-24h). Half of the plants from each group were treated with imazamox (5 mg L^−1^, black symbols). In each group, the other halves were not treated with herbicide and were used as the controls (white symbols) for the imazamox-treated plants. Total free amino acids (**A**) branched-chain (**B**) aromatic (**C**) acidic (**D**) and amide (**E**) amino acid contents. Values represent the mean ± SE (*n* = 4 biological replicates). Significant variations are marked with * for differences between control and imazamox-treated plants (*t*-Test, *p* < 0.05) at a given day. See Supporting Information Table S1 for the two-way ANOVA results.

The effects on free amino acid profile were studied in the No-Hypoxia and Hypoxia-24h groups (Figure 5) by monitoring five physiological parameters previously described to be affected by ALS inhibitors: free amino acid content, BCAA, aromatic, acidic, and amide amino acid contents [6,10,14,27]. As ALS -inhibitors cause a general increase in the content of free amino acids that could mask the specific changes in each absolute content [27,28], BCAA, aromatic, acidic and amide amino acids are shown as their relative content in terms of percentage of total amino acid content (Figure 5).

As expected, IMX treatment provoked an increase in the free amino acid pool in both groups of plants (Figure 5A). Nevertheless, the increase was significant earlier (day 1) when plants were not subjected to the hypoxic treatment. The relative content of BCAA was lower in plants treated with the herbicide in both groups, but this effect was abolished after 7 days of treatment if plants were subjected to the hypoxic treatment (Figure 5B). Aromatic amino acid content (sum of phenylalanine, tyrosine, and tryptophan) was not significantly affected by the herbicide or the hypoxic treatment (Figure 5C). Acidic amino acid content (the sum of glutamic and aspartic acids) decreased after 3 days of IMX treatment only when plants were not subjected to hypoxia before herbicide application (Figure 5D). Contrary to expectations, a general increase in the amide amino acid content was not detected (the sum of glutamine and asparagine) (Figure 5E).

**Figure 6:**
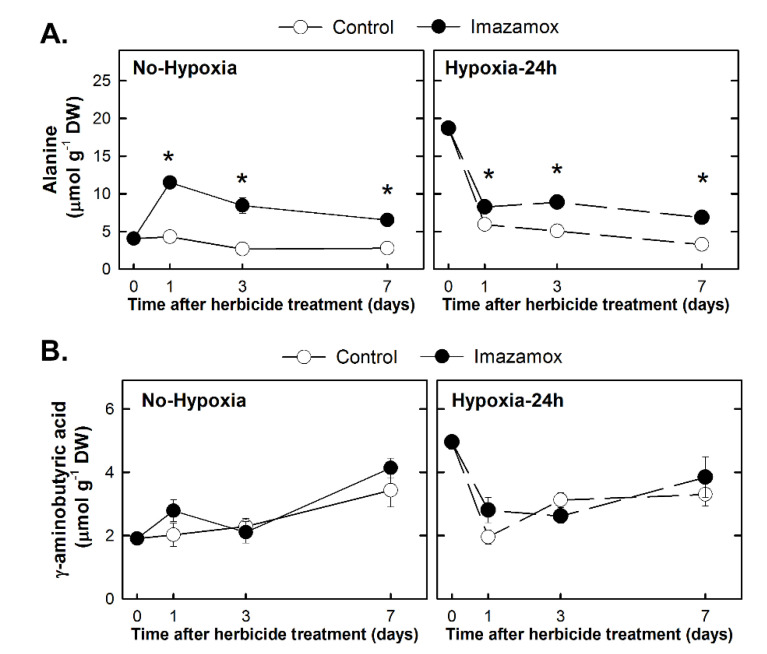
Alanine (**A**) and γ-aminobutyric acid (GABA; **B**) contents in the root of pea plants. Pea plants were not treated with hypoxia before herbicide application (Group No-Hypoxia) or were treated with hypoxia for 24 h before herbicide application (Group Hypoxia-24h). Half of the plants from each group were treated with imazamox (5 mg L^−1^, black symbols). In each group, the other halves were not treated with the herbicide and were used as the controls (white symbols) for the imazamox-treated plants. Values represent the mean ± SE (*n* = 4 biological replicates). Significant variations are marked with * for differences between control and imazamox-treated plants (*t*-Test, *p* < 0.05) at a given day. See Supporting Information Table S1 for the two-way ANOVA results.

Alanine and γ-aminobutyric acid (GABA) are two amino acids that usually accumulate under low-oxygen conditions. Their content was evaluated in both the No-Hypoxia and Hypoxia-24h groups (Figure 6). After 24 h of hypoxia, the content of GABA and alanine increased, thus, plants from the Hypoxia-24h group presented high levels of alanine and GABA at the onset of the herbicide treatment. Exposure to hypoxia did not change the effect of the herbicide, with the pattern of Alanine and GABA contents being similar in both groups: alanine content was accumulated in herbicide-treated plants over the entire time of study and GABA content was not modified by the herbicide.

## 3. Discussion

The oxygen concentration in the nutrient solution measurements and the evaluation of the enzymes involved in the ethanol fermentation validated the experimental design since the desired conditions were obtained. The oxygen concentration in the nutrient solution of the plants exposed to hypoxia before herbicide application drastically decreased: it was about 30–40% the day of herbicide application (day 0) (Figure 1). In addition, alanine and GABA were accumulated in these plants (Figure S1), which is a typical response found in plants exposed to hypoxia. On the other hand, at day 0, the activities and protein contents of PDC and ADH of the plants from the Hypoxia-24h and Hypoxia-48h groups were much higher than the activities in the non-hypoxic plants, confirming that ethanol fermentation in the roots was already induced when the herbicide was applied.

The previously described effects on ethanol fermentation, triggered as a consequence of ALS inhibition [11,13,14,15], were observed in the IMX-treated plants: increased in vitro activity of PDC (Figure 2A), higher ADH activity detected by native gel (Figure 2B), and accumulation of both PDC and ADH proteins (Figure 2C). In addition, native gel showed that the anodic isozyme demonstrated less induction than the other two bands after IMX treatment, as was described before for other ALS inhibitors [13]. Different induction pattern was described for anaerobically stressed cotton, from which the most anodic isozyme (ADH2-ADH2) was induced more than the other two [29].

In concordance with the growth arrest previously described in several plant species (e.g., soybean, pea, rice) as a consequence of ALS inhibition [4,12,30,31], the results obtained in this experiment also evidenced that IMX provoked a decrease in both shoot and root elongation (Figure 3). Oxygen availability also limits the growth of the tissues [18], and root growth of the plants subjected for 48 h to hypoxia was restricted for 3 days after returning to aerobic conditions (Figure 3C). It was not possible to detect a different effect of the herbicide on root growth depending if plants were exposed to hypoxia or not, because IMX arrested the root growth completely in the three groups.

Carbohydrates were accumulated in the roots as a consequence of ALS inhibition (Figure 4) as has been described before [8,9,10,11,14,32,33]. The accumulation in roots was due to a lack of use of available sugars because growth was arrested (Figure 3). This carbohydrate accumulation has not been previously related to a change in total respiration rates or cytochrome respiratory capacity, but an increase in AOX capacity was observed in a previous experiment with the same plant material but with other ALS inhibitors [9]. These results suggest that ALS inhibition induce the less-efficient ATP-producing pathways of fermentation and alternative respiration, while growth is arrested and carbohydrates are not consumed.

Although carbohydrate accumulation was detected after IMX treatment in hypoxic plants (groups Hypoxia-24h and Hypoxia-48h), the total soluble accumulation was detected later than in the non-hypoxic plants and the starch accumulation was lower than in the non-hypoxic plants (Figure 4). Although it can be argued that hypoxia incurred a carbon deficit which delayed the later carbohydrate accumulation due to herbicide, this was not true, as previous findings revealed that carbohydrate shortage was not detected in the pea roots subjected to hypoxia to 1 or 3 days (Figure 4 in [34]). So, the lower and later carbohydrate accumulation induced by the herbicide in hypoxic plants compared to non-hypoxic suggests an alleviation of this physiological effect on carbon metabolism if plants are subjected to hypoxia before herbicide application.

Moreover, regarding the nitrogen metabolism, an increase in the total free amino acid content and several changes in individual amino acids were detected after IMX treatment (Figure 5). Free amino acid accumulation has been previously observed in plants treated with different ALS inhibitors [6,7,35] and it has been proposed to occur due to an increased protein turnover [36], which can be related to an altered, proteolytic profile [7]. Contrary to previous studies [10,27], aromatic amino acid content was not affected by the ALS-inhibitors.

BCAA relative content was decreased after IMX treatment during all the period of study while in previous studies the decrease was transitory, abolished after 4 days [27,37,38] and followed by an increase. The initial decrease of the BCAA and the following increase agree with the proteolysis discussed above.

Acidic and amide amino acid contents showed a tendency to the pattern expected, increase of amides and decrease of acidic amino acids [10,27], although the changes were not consistent during all the time of study. In general, the proportion of the two acidic amino acids was lower in herbicide-treated plants than in the control, and the proportion of their amides was higher in herbicide-treated plants than in control plants (Figure 5). This behaviour shows that the plant develops a progress in nitrogen storage from amino acids in the acidic form to the amide form. These coordinated variations are difficult to explain, and it is not possible finally to establish if there are key enzymes under common control, if such modulation involves parameters such as glutamine/glutamate [39] or if there is a general control of C/N metabolism just by controlling the ‘glutamine plus asparagine/glutamate plus aspartate’ proportion.

Interestingly, several of these effects described after the herbicide on the amino acid profile were attenuated if ethanol fermentation was induced before herbicide application: BCAA decrease after 7 days was abolished and amide amino acid increase and acidic amino acid decrease were attenuated.

Alanine was accumulated at the onset of the herbicide treatments in plants subjected to hypoxia (Figure 6A). The production of alanine from pyruvate through alanine amino-transferase is another quite important pathway related to fermentation and lack of oxygen [40]. Alanine was accumulated in herbicide treated plants both in hypoxic and non-hypoxic plants, as was detected in Arabidopsis plants treated with foramsulfuron, [37] or pea roots treated with imazethapyr [34], other two ALS inhibitors and which can be related to the previously detected induction of alanine amino-transferease [11,14]. The non-protein amino acid GABA (Figure 6B) was accumulated after 48 h of hypoxia. It is a metabolite that is usually accumulated under stress situations, such as anoxia [41,42].

Plant death following ALS inhibition, has been associated with an impairment of carbon and nitrogen metabolism, only detected after ALS inhibition and not detected after inhibition of other enzymes of the BCAA pathway. Thus, the different herbicidal efficacy observed could be associated with a different carbon/nitrogen metabolism imbalance [43]. The results of this study evidence that hypoxia alleviates the impairment of carbon and nitrogen metabolism induced by ALS inhibitors, suggesting that an induced fermentative metabolism alleviates the herbicidal effects on the physiology of treated plants.

Several other approaches have tried to examine the significance of the induction of fermentation in response to the ALS-inhibiting herbicides to evaluate if it is part of the plant defense against herbicides or by contrast, it contributes to their chemical toxicity. Unfortunately, until now, divergent results have been reported. On one hand, alleviation of the effects of ALS inhibitor herbicides on different parameters (carbohydrate and amino acid accumulation) was observed in the *adh1* mutant supporting that fermentation contributes to the herbicide action [14]. On the other hand, mutants with reduced fermentative potential exhibited higher sensitivity to herbicide treatments [44] and plants overexpressing the PDH bypass were less sensitive to ALS-inhibitors [45], supporting the hypothesis that induction of PDC contributes to herbicide stress tolerance. The present study shows that fermentation induction at the moment of herbicide application decreases the herbicide physiological action and supports the second of the two hypothesis proposed about the role of fermentation in the mode of action of ALS-inhibitors: the induction of ethanol fermentation in treated plants is part of the plant defense mechanism after herbicide.

## 4. Materials and Methods

### 4.1. Plant Material and Treatment Application

*Pisum sativum* L. cv. snap sugar boys where surface sterilized and grown under controlled conditions [9]. To prevent roots from hypoxia, the nutrient solution was continuously aerated. When the plants were 12-days-old, they were separated in three groups (Table 1). In order to obtain low-oxygen conditions for the induction of fermentation, the aeration was removed in one of the groups for 48 h (named Hypoxia-48h), and for 24 h in another group (named Hypoxia-24h). After the 48 h or 24 h the tanks were again aerated, this day was considered as the day 0. The other group was maintained continuously aerated (named No-Hypoxia). The nutrient solution was replaced the day 0 in all the tanks and the herbicide was applied to half of the tanks from the group Hypoxia-48h (named 48h-IMX), half of the plants from the group Hypoxia-24h (named 24h-IMX), and to half of the tanks from the group No-Hypoxia (named 0-IMX). The herbicide was applied to the nutrient solution as commercial formulation at a final concentration of 5 mg active ingredient L^−1^ (16.33 μM) of IMX (Pulsar^®^40, BASF Española SA, Barcelona, Spain). The other half of the plants from the groups Hypoxia-48h, Hypoxia-24h and No-Hypoxia were not treated with herbicide (named as 48h-C, 24h-C and 0-C, respectively) and were used as the control for the comparison with the herbicide-treated plants of the corresponding group. The experiment was performed in duplicate. The applied herbicide doses provoked plant death in 20 days.

For the analytical measurements, intact root samples were taken at day 0, before herbicide application, and at days 1, 3 and 7 after IMX application, this time points were chosen in order to allow us to evaluate physiological and biochemical plant responses induced by the herbicide, but not directly resulting from cell death.

Plant material was immediately frozen in liquid nitrogen and stored at −80 °C for further analysis. Later, frozen samples were ground under liquid nitrogen using a Retsch mixer mill (MM200, Retsch^®^, Haan, Germany), the required amount of tissue for each analysis was separated and stored at −80 °C. Some fresh material was dried for 48 h at 80 °C in order to obtain the fresh weight/dry weight ratio.

### 4.2. PDC and ADH Enzymatic Activities

Briefly, PDC and ADH activities were measured in a spectrophotometer monitoring NADH consumption or formation at 340 nm, respectively, as described before [11].

In gel, ADH activity was measured in desalted extracts obtained from pea roots in a ratio of 0.1 g FW/2.5 mL extraction buffer. Native electrophoresis was run in a 12.5% polyacrylamide gel (Phast Gel^®^ Homogeneous 12.5% in 0.112 M Acetate, 0.112 M Tris (pH 6.4)) at 4 °C in a Phast SystemTM (Pharmacia, LKB, Biotechnology AB, Uppsala, Sweden). Phast Gel^®^ Buffer Strips Native (0.88 M L-Alanine, 0.25 M Tris (pH 8.8)) were used for the electrophoresis. In each line, 1.95 μg of protein was loaded.

ADH specific staining was performed as described in a previous study [26] with minor modifications. The gel was incubated in darkness for 15 min in a solution composed of 25 mM Tris-Cl (pH 8), 0.8% (*v/v*) ethanol, 0.144 mM nitro blue tetrazolium, 0.65 mM phenazine methosulfate, and 0.24 mM NAD+.

### 4.3. PDC and ADH Immunoblotting

PDC and ADH protein immunoblot assay was performed according to standard techniques, as described in a previous study [46]. PDC and ADH antibodies were used at dilutions of 1:1000 and 1:500, respectively. Goat anti-rabbit IgG alkaline phosphatase (Sigma-Aldrich) was used as the secondary antibody at a dilution of 1:20,000, and cross-reacting protein bands were visualized using the Amplified Alkaline Phosphatase Goat Anti-Rabbit Immun-Blot^®^ Assay Kit (Bio-Rad Inc., Hercules, CA, USA), according to the manufacturer’s instructions. The intensity of the bands was quantified using a GS-800 densitometer (Bio-Rad Inc., Hercules, CA, USA).

### 4.4. Metabolites Determination

#### 4.4.1. Amino Acids

The extraction of amino acids from pea roots was performed in HCl. After protein precipitation, amino acid concentrations were measured in the supernatant using capillary electrophoresis equipped with a laser-induced fluorescence detector, as previously described [27].

#### 4.4.2. Total Soluble Sugars and Starch

The glucose, fructose, and sucrose (total soluble sugars) concentrations were determined in ethanol-soluble extracts, and the ethanol-insoluble residue was extracted for starch analysis. Starch and soluble sugar concentrations were determined using high-performance capillary electrophoresis, as previously described [8].

### 4.5. Statistical Analysis

All analyses were performed using four biological replicates from two independent experiments. The mean was used as a measure of central tendency and the standard error (SE) as a measure of dispersion.

First, the data of the three different groups (No-Hypoxia, Hypoxia-24h, and Hypoxia-48h) were compared independently. The herbicide-treated plants were compared with their respective controls for each sampling day using the Student’s *t*-Test for the Significance of the Difference between the Means of Two Independent Samples. Second, for all the studied parameters, a two-way analysis of variance (ANOVA) was performed to examine the influence of the studied variables (hypoxia and herbicide application) and their possible interaction. When the results were expressed in percentages, the data were previously transformed according to the following formula: *arcsin*√*x*/100. The statistical analysis was conducted at a significance level of 5% (*p* < 0.05) and the statistical program used was IBM SPSS Statistics (v.22).

## 5. Conclusions

In this study, an original approach was used to unravel the role of fermentation induction after ALS-inhibiting herbicides, by evaluating the effect of applying hypoxia before the herbicide on the typical physiological markers in herbicide-treated plants.

There are several physiological effects widely described after ALS inhibition, that have been also detected in pea roots in this study: carbohydrate accumulation, decrease in BCAA and acidic amino acids and increase in amide amino acids. The changes in these physiological markers was lower or later if the plants were subjected to hypoxia before treatment application. This decrease of the herbicide action with hypoxia supports the hypothesis that fermentation induction by ALS inhibitors is a plant defense mechanism counteracting the physiological effect of the herbicide.

## Figures and Tables

**Table 1:** Summary of the treatments.

Group	Abbreviation	Treatment Description
No-Hypoxia	0-C	No treatment was applied.
	0-IMX	Application of 5 mg L^−1^ of imazamox at day 0.
Hypoxia-24h	24h-C	The aeration was removed for 24 h before the day 0. Aeration was again placed at day 0 until the end of the experiment. No herbicide was applied.
	24h-IMX	The aeration was removed for 24 h before the day 0. The aeration was again placed at day 0 until the end of the experiment. At day 0 imazamox was applied at a final concentration of 5 mg L^−1^.
Hypoxia-48h	48h-C	The aeration was removed for 48 h before the day 0. Aeration was again placed at day 0 until the end of the experiment. No herbicide was applied.
	48h-IMX	The aeration was removed for 48 h before the day 0. Aeration was again placed at day 0 until the end of the experiment. The day 0 imazamox was applied at a final concentration of 5 mg L^−1^.

## Supplementary Materials

The following are available online at https://www.mdpi.com/2223-7747/9/8/981/s1, Figure S1: Original blots and band intensity of the Native PAGE for alcohol dehydrogenase, Figure S2: Original blots and band intensity of the immunoblot detection of pyruvate decarboxylase, Figure S3: Original blots and band intensity of the immunoblot detection of alcohol dehydrogenase, Table S1: Results of the two-way analysis of variance.
