# Supplementary material for: Hypoxic Treatment Decreases the Physiological Action of the Herbicide Imazamox on Pisum sativum Roots
Source: Plants (Basel). 2020 Aug 3;9(8):981. doi: 10.3390/plants9080981 (PMC7464988; doi:10.3390/plants9080981)
Supplement: Supplementary file 1 [file plants-09-00981-s001.pdf]

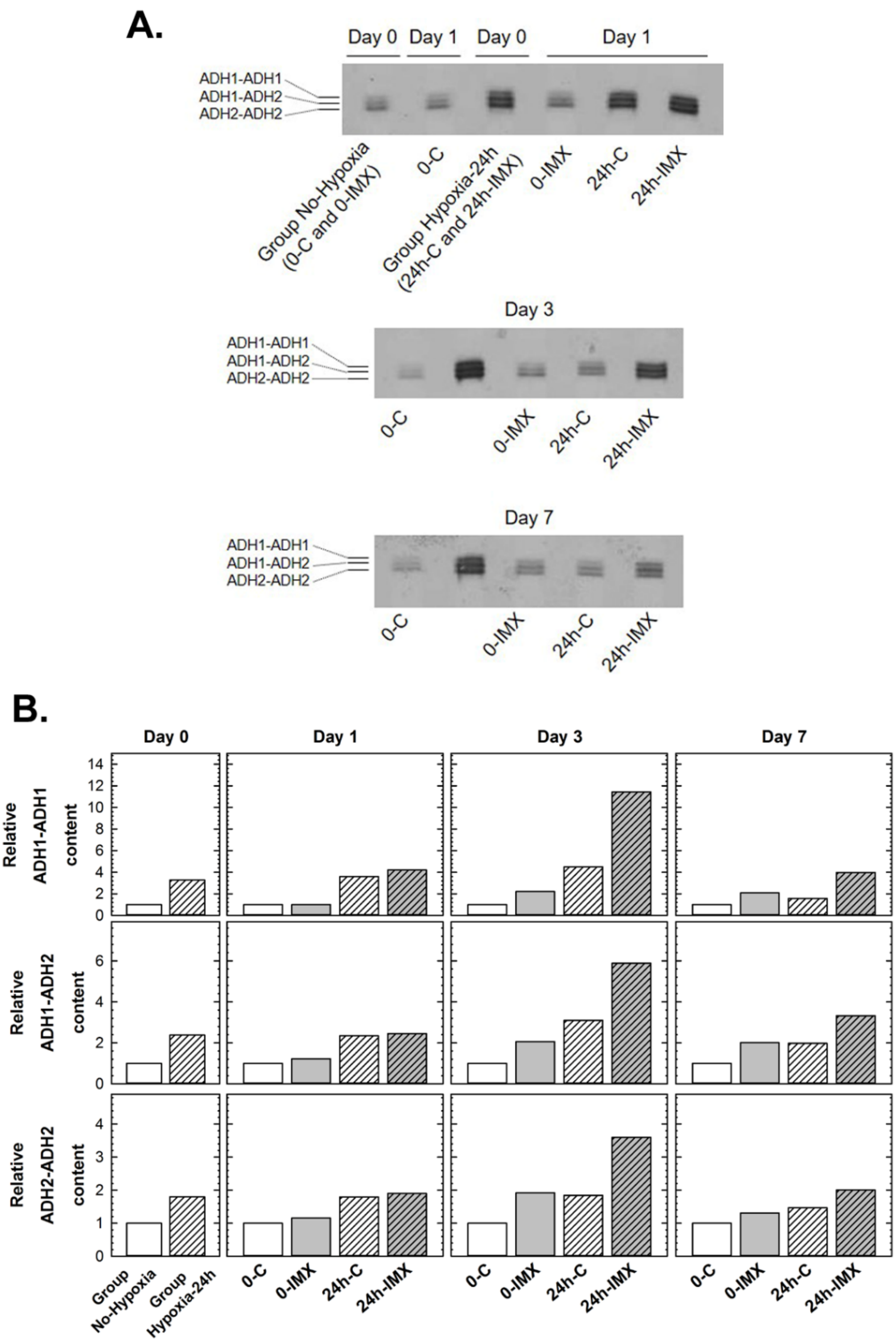

Figure S1. Original blots (A) and band intensity (B) of the Native PAGE for alcohol dehydrogenase (ADH) activity in pea roots on days 0, 1, 3, and 7 after imazamox application. Analyses of band intensity on blots are presented in graphs as the relative ratio of the 0-C treatment of the corresponding day. Pea plants were not treated with hypoxia before herbicide application (the No-Hypoxia group) or were treated with hypoxia for 24 h before herbicide application (the Hypoxia-24h group). Half of the plants from each group were treated with imazamox (5 mg L^{-1}). The others were not treated with herbicide and were used as controls. The different treatments were named with a combination of two codes: the first code refers to the hours of hypoxia (0 or 24 h), and the second one indicates if plants were treated with imazamox (IMX) or were controls (C): 0-C, 0-IMX, 24h-C, 24h-IMX. Each lane contained 1.75 μg of protein.

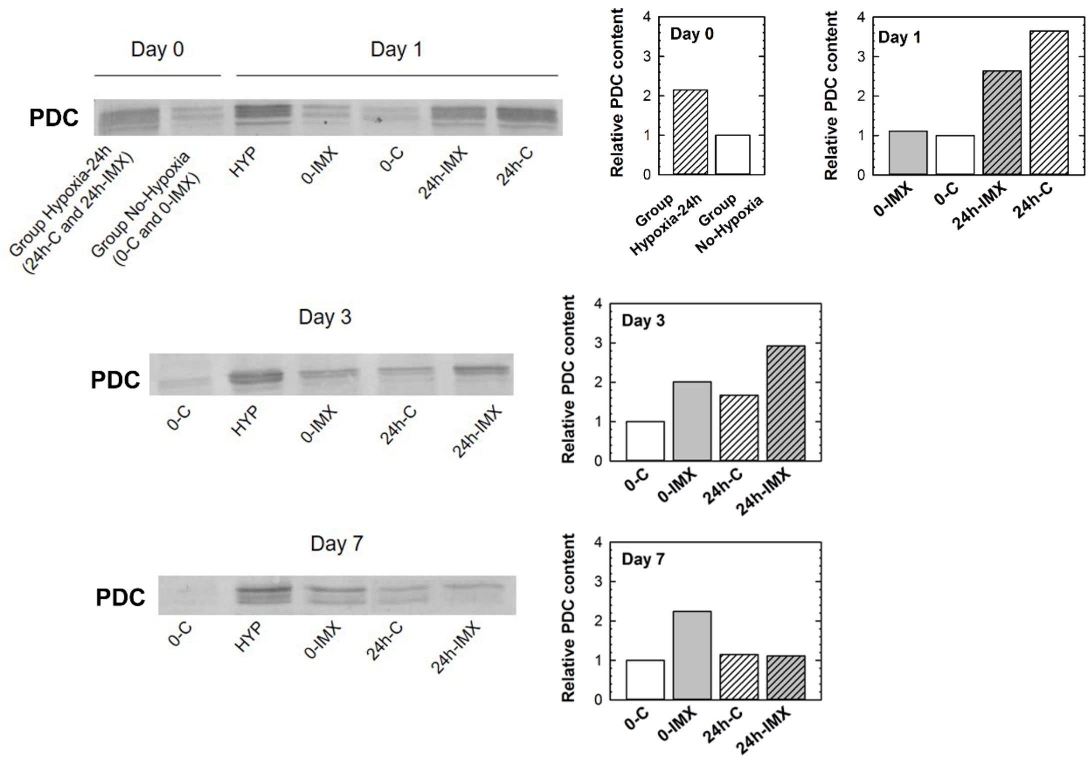

Figure S2. Original blots (left) and band intensity (right) of the immunoblot detection of pyruvate decarboxylase (PDC) on days 0, 1, 3, and 7 after imazamox application. Analyses of band intensity on blots are presented in graphs as the relative ratio of the 0-C treatment of the corresponding day. Pea plants were not treated with hypoxia before herbicide application (the No-Hypoxia group) or were treated with hypoxia for 24 h before herbicide application (the Hypoxia-24h group). Half of the plants from each group were treated with imazamox (5 mg L^{-1}). The others were not treated with herbicide and were used as controls. The different treatments were named with a combination of two codes: the first code refers to the hours of hypoxia (0 or 24 h), and the second one indicates if plants were treated with imazamox (IMX) or were controls (C): 0-C, 0-IMX, 24h-C, 24h-IMX. Plants exposed to hypoxia during all the course of experiment (HYP) were used as positive control. Each lane contained 30 µg of protein.

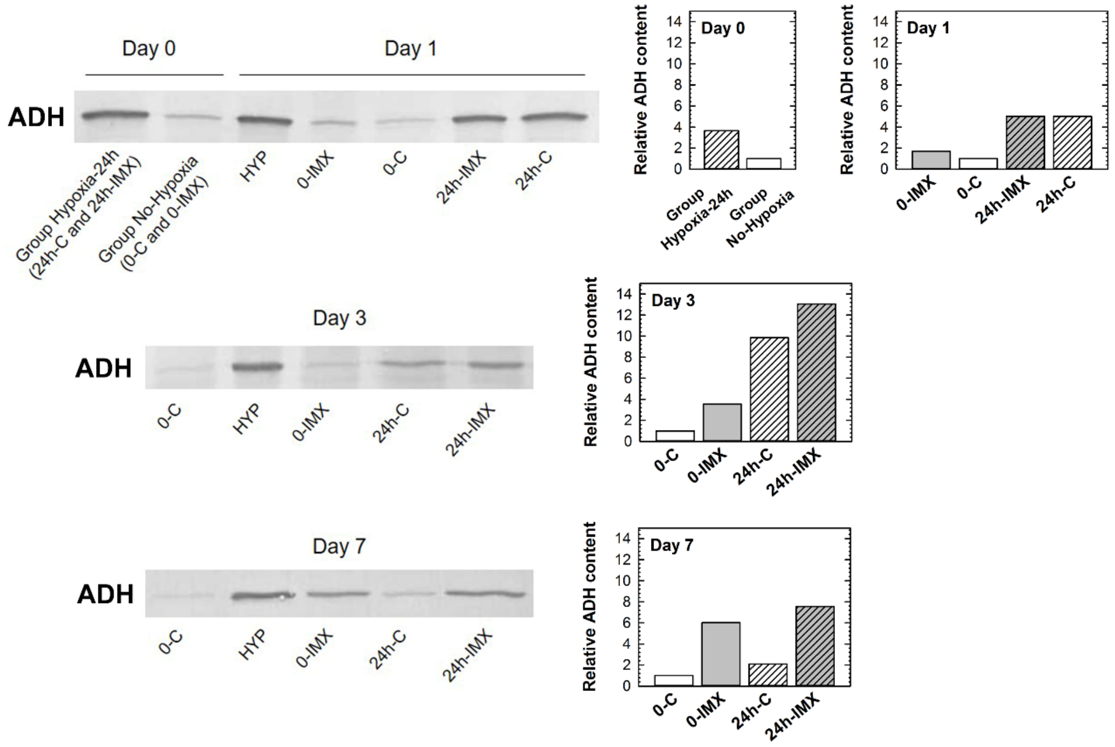

Figure S3. Original blots (left) and band intensity (right) of the immunoblot detection of alcohol dehydrogenase (ADH) on days 0, 1, 3, and 7 after imazamox application. Analyses of band intensity on blots are presented in graphs as the relative ratio of the 0-C treatment of the corresponding day. Pea plants were not treated with hypoxia before herbicide application (the No-Hypoxia group) or were treated with hypoxia for 24 h before herbicide application (the Hypoxia-24h group). Half of the plants from each group were treated with imazamox (5 mg L^{-1}). The others were not treated with herbicide and were used as controls. The different treatments were named with a combination of two codes: the first code refers to the hours of hypoxia (0 or 24 h), and the second one indicates if plants were treated with imazamox (IMX) or were controls (C): 0-C, 0-IMX, 24h-C, 24h-IMX. Plants exposed to hypoxia during all the course of experiment (HYP) were used as positive control. Each lane contained 30 μg of protein.

Table S1. Results of the two-way analysis of variance. Significance level p < 0.05 (* = p < 0.05; ns = not significant). ADH, alcohol dehydrogenase; BCAA, branched-chain amino acids; GABA, γ-aminobutyric acid; PDC, pyruvate decarboxylase.

Figure 2. PDC and ADH activities							
		**Day 1**		**Day 3**		**Day 7**	
**PDC activity**	Herbicide	0.169	ns	0.988	ns	0.017	*
	Hypoxia	0.003	*	0.333	ns	0.450	ns
	Herbicide x Hypoxia	0.161	ns	0.051	ns	0.412	ns
**ADH activity**	Herbicide	0.632	ns	0.682	ns	0.205	ns
	Hypoxia	0.001	*	0.146	ns	0.451	ns
	Herbicide x Hypoxia	0.081	ns	0.757	ns	0.598	ns

Figure 3. Growth							
		**Day 1**		**Day 3**		**Day 7**	
**Shoot length**	Herbicide	0.515	ns	0.738	ns	0.087	ns
	Hypoxia	0.082	ns	0.631	ns	0.066	ns
	Herbicide x Hypoxia	0.144	ns	0.681	ns	0.392	ns
**Root length**	Herbicide	0.531	ns	0.014	*	0.000	*
	Hypoxia	0.012	*	0.006	*	0.064	ns
	Herbicide x Hypoxia	0.963	ns	0.229	ns	0.219	ns

Figure 4. Carbohydrate content							
		**Day 1**		**Day 3**		**Day 7**	
**Total soluble sugars content**	Herbicide	0.001	*	0.000	*	0.000	*
	Hypoxia	0.464	ns	0.018	*	0.809	ns
	Herbicide x Hypoxia	0.078	ns	0.005	*	0.761	ns
**Starch content**	Herbicide	0.034	*	0.000	*	0.008	*
	Hypoxia	0.151	ns	0.996	ns	0.364	ns
	Herbicide x Hypoxia	0.193	ns	0.625	ns	0.080	ns

Figure 5. Amino acid content								
		Day 1		Day 3		Day 7		
**Total free amino acid content**	Herbicide	0.064	ns	0.001	*	0.001	*
	Hypoxia	0.522	ns	0.457	ns	0.273	ns
	Herbicide x Hypoxia	0.746	ns	0.607	ns	0.823	ns
**BCAA content**	Herbicide	0.000	*	0.000	*	0.023	*
	Hypoxia	0.024	*	0.724	ns	0.196	ns
	Herbicide x Hypoxia	0.162	ns	0.300	ns	0.166	ns
**Aromatic amino acid content**	Herbicide	0.076	ns	0.359	ns	0.060	ns
	Hypoxia	0.121	ns	0.799	ns	0.708	ns
	Herbicide x Hypoxia	0.740	ns	0.359	ns	0.208	ns
**Acidic amino acid content**	Herbicide	0.266	ns	0.045	*	0.640	ns
	Hypoxia	0.101	ns	0.269	ns	0.178	ns
	Herbicide x Hypoxia	0.925	ns	0.059	ns	0.045	*
**Amide amino acid content**	Herbicide	0.094	ns	0.126	ns	0.000	*
	Hypoxia	0.730	ns	0.818	ns	0.810	ns
	Herbicide x Hypoxia	0.165	ns	0.818	ns	0.577	ns

Figure 6. Alanine and GABA content							
		Day 1		Day 3		Day 7	
**Alanine content**	Herbicide	0.000	*	0.000	*	0.000	*
	Hypoxia	0.250	ns	0.003	*	0.457	ns
	Herbicide x Hypoxia	0.003	*	0.011	*	0.887	ns
**GABA content**	Herbicide	0.036	*	0.213	ns	0.244	ns
	Hypoxia	0.940	ns	0.026	*	0.686	ns
	Herbicide x Hypoxia	0.889	ns	0.562	ns	0.873	ns
